# SIRT1 modulates cell cycle progression by regulating CHK2 acetylation−phosphorylation

**DOI:** 10.1038/s41418-019-0369-7

**Published:** 2019-06-17

**Authors:** Wenyu Zhang, Yanling Feng, Qiqiang Guo, Wendong Guo, Hongde Xu, Xiaoman Li, Fei Yi, Yi Guan, Nanxi Geng, Pingyuan Wang, Longyue Cao, Brian P. O’Rourke, Juhyeon Jo, Jiyun Kwon, Ruihong Wang, Xiaoyu Song, In Hye Lee, Liu Cao

**Affiliations:** 1 Institute of Translational Medicine, Key Laboratory of Cell Biology of Ministry of Public Health, and Key Laboratory of Medical Cell Biology of Ministry of Education, Liaoning Province Collaborative Innovation Center of Aging Related Disease Diagnosis and Treatment and Prevention, China Medical University, No. 77, Puhe Road, Shenyang North New Area, Shenyang, 110122 Liaoning China; 2 Department of Cell Biology, Key Laboratory of Cell Biology of Ministry of Public Health, and Key Laboratory of Medical Cell Biology of Ministry of Education, China Medical University, No. 77, Puhe Road, Shenyang North New Area, Shenyang, 110122 Liaoning China; 3 Center for Molecular Medicine, National Heart, Lung, and Blood Institute, National Institutes of Health, Bethesda, MD 20892 USA; 4 Department of Medicine (Cardiology), Wilf Family Cardiovascular Research Institute, Albert Einstein College of Medicine, Bronx, NY 10461 USA; 5 Department of Physiology and Biophysics, Albert Einstein College of Medicine, Bronx, NY 10461 USA; 6 Department of Life Science, College of Natural Science Office #106, Science building C, Ewha Womans University 52, Ewhayeodae-gil, Seodaemun-gu, Seoul, 03760 South Korea; 7 Faculty of Health Science, University of Macau, Macau, China

**Keywords:** Cell biology, Epigenetics

## Abstract

Both the stress-response protein, SIRT1, and the cell cycle checkpoint kinase, CHK2, play critical roles in aging and cancer via the modulation of cellular homeostasis and the maintenance of genomic integrity. However, the underlying mechanism linking the two pathways remains elusive. Here, we show that SIRT1 functions as a modifier of CHK2 in cell cycle control. Specifically, SIRT1 interacts with CHK2 and deacetylates it at lysine 520 residue, which suppresses CHK2 phosphorylation, dimerization, and thus activation. SIRT1 depletion induces CHK2 hyperactivation-mediated cell cycle arrest and subsequent cell death. In vivo, genetic deletion of *Chk2* rescues the neonatal lethality of *Sirt1*^*−/−*^ mice, consistent with the role of SIRT1 in preventing CHK2 hyperactivation. Together, these results suggest that CHK2 mediates the function of SIRT1 in cell cycle progression, and may provide new insights into modulating cellular homeostasis and maintaining genomic integrity in the prevention of aging and cancer.

## Introduction

CHK2 is the mammalian homolog of *Saccharomyces cerevisiae* Rad53 and *Schizosaccharomyces pombe* Cds1, yeast kinases that are active in that organism’s DNA damage response (DDR), acting as critical regulators of genome integrity checkpoints [1]. Previous studies have suggested that CHK2 is a key component in several molecular processes involved in DNA structural modification, cell cycle progression, cell stemness maintenance, circadian clock control, and DDR [2–4]. Disruption of these checkpoints may cause genomic instability and cell death, and contribute to tumor formation [5]. Likewise, increasing lines of evidence suggest that CHK2 serves as an essential surveillant of cell survival and various pathophysiological processes, including aging and cancer [6, 7]. Studies indicate that phosphorylation of CHK2 is a versatile means to specifically and rapidly modulate its activity and to further define its biological functions [4]. Nevertheless, little is known about other post-translational modifications (PTMs) involved in CHK2 activation.

Recent evidence suggests that protein acetylation is a widely employed PTM that can alter a protein’s ability to bind DNA, undergo activation/inactivation, participate in PPI, alter subcellular localization, or regulate stability and degradation [8, 9]. Reversible acetylation is known to be catalyzed by a group of histone acetyltransferases (HATs) and histone deacetylases (HDACs) [10]. There is now accumulating evidence for the role of acetylation in fine-tuning non-histone protein function, as well as modulating a diverse array of cellular functions in order to maintain mammalian cell homeostasis.

Among the sirtuin family of protein deacetylases (SIRT1–7), whose catalytic activity is uniquely dependent on NAD^+^, SIRT1 shares the highest mammalian homology with the yeast silent information regulator 2 [11, 12]. As the most well-studied sirtuin, SIRT1 has been implicated in many physiological and pathophysiological processes, including the circadian clock, neuronal protection, caloric restriction, cell cycle arrest, apoptosis, glucose and lipid metabolism, cellular senescence, and cancer [13–19]. The diverse range of deacetylation substrates of SIRT1 confers its multiple biological functions. For example, SIRT1 can act as either a promoter or a suppressor in tumorigenesis depending on the specific context of its diverse downstream effectors [20]. Previous studies have shown that genetic mutation or deletion of the *Sirt1* gene result in perinatal lethality [21, 22]. However, the underlying mechanism remains poorly determined. It is reported that SIRT1 deficiency could activate p53 to suppress cell survival by promoting its hyperacetylation [23], whereas simultaneous depletion of *p53* failed to rescue the lethality of *Sirt1*-deficient mice, suggesting that hyperactivation of p53 is not the main reason for the lethality of mice lacking *Sirt1* [22].

Here we find that SIRT1 and P300 regulate CHK2 acetylation, with lysine 235 and 520 as the primarily acetylated residues. Furthermore, CHK2 acetylation at the K520 site contributes to its dimerization and activation. Importantly, we discovered that defects in cellular homeostasis caused by SIRT1 depletion are at least partially through hyperactivation of CHK2, as evidenced by a mouse model wherein the neonatal lethality of *Sirt1*-deficient mice is rescued by genetic disruption of *Chk2*. Therefore, our study reveals that a SIRT1-regulated acetylation pathway mediates CHK2 activation and suggests that the SIRT1–CHK2 axis is required for genomic integrity and cellular homeostasis.

## Results

### SIRT1 interacts with and suppresses CHK2 phosphorylation

**Fig. 1:**
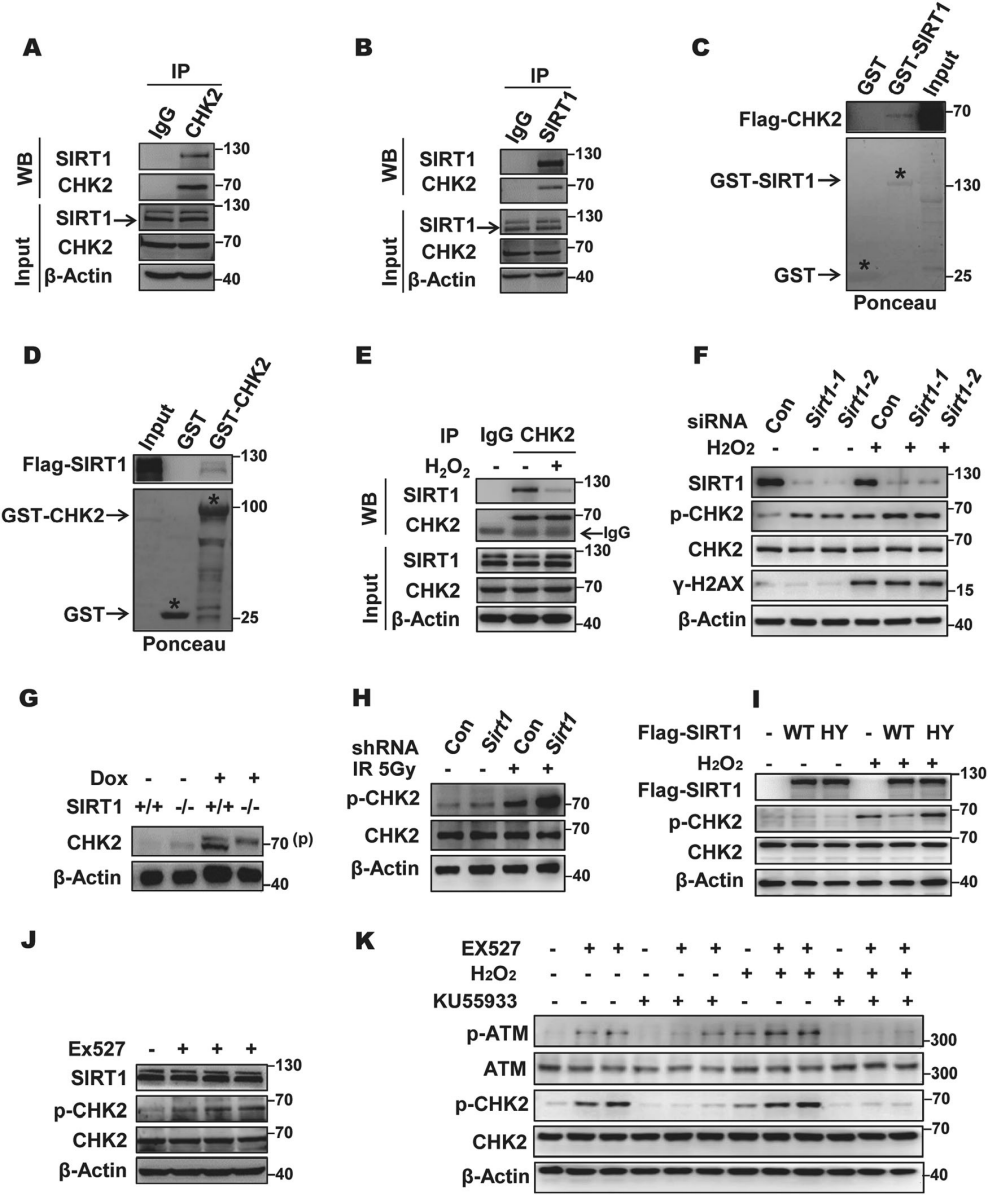
SIRT1 binds to and negatively regulates CHK2 phosphorylation. **a**, **b** SIRT1 interacts with CHK2 in vivo. HEK293 cell lysates were subjected to immunoprecipitation with control IgG, anti-SIRT1 (**a**), or anti-CHK2 (**b**) antibodies. The immunoprecipitates were then blotted with the indicated antibodies. **c**, **d** SIRT1 interacts with CHK2 in vitro. Recombinant human SIRT1 (**h**) or CHK2 (**i**) was incubated with bacterially expressed GST-CHK2 (**h**) and GST-SIRT1 (**d**) for 1 h at 30 °C. **e** Hydrogen peroxide (H_2_O_2_) treatment decreased the binding of CHK2 to SIRT1. Endogenous immunoprecipitation was performed with control IgG or anti-CHK2 antibodies in HEK293 cells treated with 200 μM or without H_2_O_2_ for 1 h. Then, the immunoprecipitates were blotted with the indicated antibodies. **f** SIRT1 knockdown increased the p-CHK2 (CHK2 phosphorylation on Threonine 68 site). Different small interfering RNAs (siRNAs) (#1 and #2) for SIRT1 were transfected into HCT116 cells treated with or without 200 μM H_2_O_2_ for 6 h. CHK2 phosphorylation was determined with western blot analysis. **g** *SIRT1*^*+/+*^ and *SIRT1*^*−/−*^ mouse embryonic fibroblasts (MEFs) were treated with or without 200 ng doxorubicin for 12 h. The p-CHK2 level was determined with western blot analysis. **h** HCT116 cells stably expressing control or *Sirt1* short hairpin RNA (shRNA) were irradiated at 5 Gy and released for 1 h. Cell lysates were subjected to western blot analysis. **i** Catalytic activity of SIRT1 is required for phosphorylation of CHK2. HCT116 cells were transfected into Flag-tagged SIRT1 wild-type (WT) or catalytically inactive mutant H363Y in the absence or presence of 100 μM H_2_O_2_. CHK2 phosphorylation on threonine 68 residue (T68) was measured by western blot. **j** Catalytic activity of SIRT1 inhibition increases CHK2 phosphorylation. HEK293 cells treated with SIRT1 inhibitor EX527 at 0.5 μM for 0, 3, 6, and 9 h were lysed and cell lysates were blotted and measured with the indicated antibodies. **k** HCT116 cells were treated with or without EX527 at 0.5 μM and Ku55933 at 10 μM for 6 h as indicated, and then cultured in the presence or absence of 100 μM H_2_O_2_ for 1 h. Total cell lysates were subjected to western blot analysis. See also Fig. S1

Although DNA damage influences SIRT1 activity and SIRT1-dependent deacetylation regulates cell cycle checkpoint proteins, it remains unknown whether CHK2 is regulated by SIRT1 [24–26]. To address this question, we first tested a possible interaction between SIRT1 and CHK2 by co-immunoprecipitation (Co-IP). As shown in Fig. 1a, b and Fig. S1A (mouse embryonic fibroblasts (MEFs)), SIRT1 immunoprecipitated with CHK2; meanwhile, reciprocal IP with CHK2 antibodies showed that CHK2 immunoprecipitated with SIRT1. Colocalization of SIRT1 and CHK2 was also detected in the nucleus by confocal microscopy (Fig. S1B). The SIRT1–CHK2 interaction was further confirmed by in vitro glutathione-*S*-transferase (GST) pull-down assays (Fig. 1c, d). Interestingly, the SIRT1–CHK2 interaction can be disrupted by hydrogen peroxide (H_2_O_2_)-induced oxidative stress (Fig. 1e and Fig. S1C).

Phosphorylation is the most well-studied PTM of CHK2, and the phosphorylation of CHK2 at threonine 68 residue (T68) is the primary signal for CHK2 activation [4]. Therefore, we next sought to explore the possible relationship between SIRT1 and CHK2 T68 threonine phosphorylation (p-CHK2, the same below). Exogenous H_2_O_2_ were used as a means to trigger p-CHK2 induction. We found that p-CHK2 was quickly elevated in response to oxidative stress, while the expression level of SIRT1 increased gradually (Fig. S1D). The increase in SIRT1 correlated with p-CHK2 level returning to baseline. Therefore, we speculated that SIRT1 may negatively regulate CHK2 phosphorylation. Indeed, RNA interference (RNAi)-mediated knockdown of SIRT1 increased the level of p-CHK2, and was effective following DNA damage (Fig. 1f and Fig. S1E). We went onto investigate whetherdoxorubicin treatment increased p-CHK2 (supershift band) levels markedly in *SIRT1*^*−/−*^ MEFs when compared to *SIRT1*^*+/+*^ MEFs (Fig. 1g). Moreover, we validated that SIRT1 also controlled p-CHK2 upon ionizing radiation (IR) treatment, indicating it is not stress-type specific (Fig. 1h and Fig. S1F). Consistent with the result, HCT116 and H1299 cells expressing wild-type (WT) SIRT1 showed lower p-CHK2 levels than those expressing the mutant SIRT1 (H363Y) in either the absence or the presence of oxidative stress (Fig. 1i and Fig. S1G). The result was further substantiated by dose-dependent expression of the WT-SIRT1 and the catalytically inactive mutant H363Y SIRT1 in cells, which showed that the suppression of CHK2 phosphorylation by SIRT1 required SIRT1 enzymatic activity (Fig. S1H). Furthermore, as shown in Fig. 1j and Fig. S1I, S1J, a time-dependent increase of p-CHK2 was also observed in cells treated with EX527, a specific chemical inhibitor of SIRT1. In addition, we showed that ATM phosphorylation on serine 1981 is required for SIRT1-mediated regulation of p-CHK2 (Fig. 1k and Fig. S1K). Together, these results demonstrate that SIRT1 is responsible for modulating CHK2 dephosphorylation.

### SIRT1 and p300 regulate an acetylation switch of CHK2

**Fig. 2:**
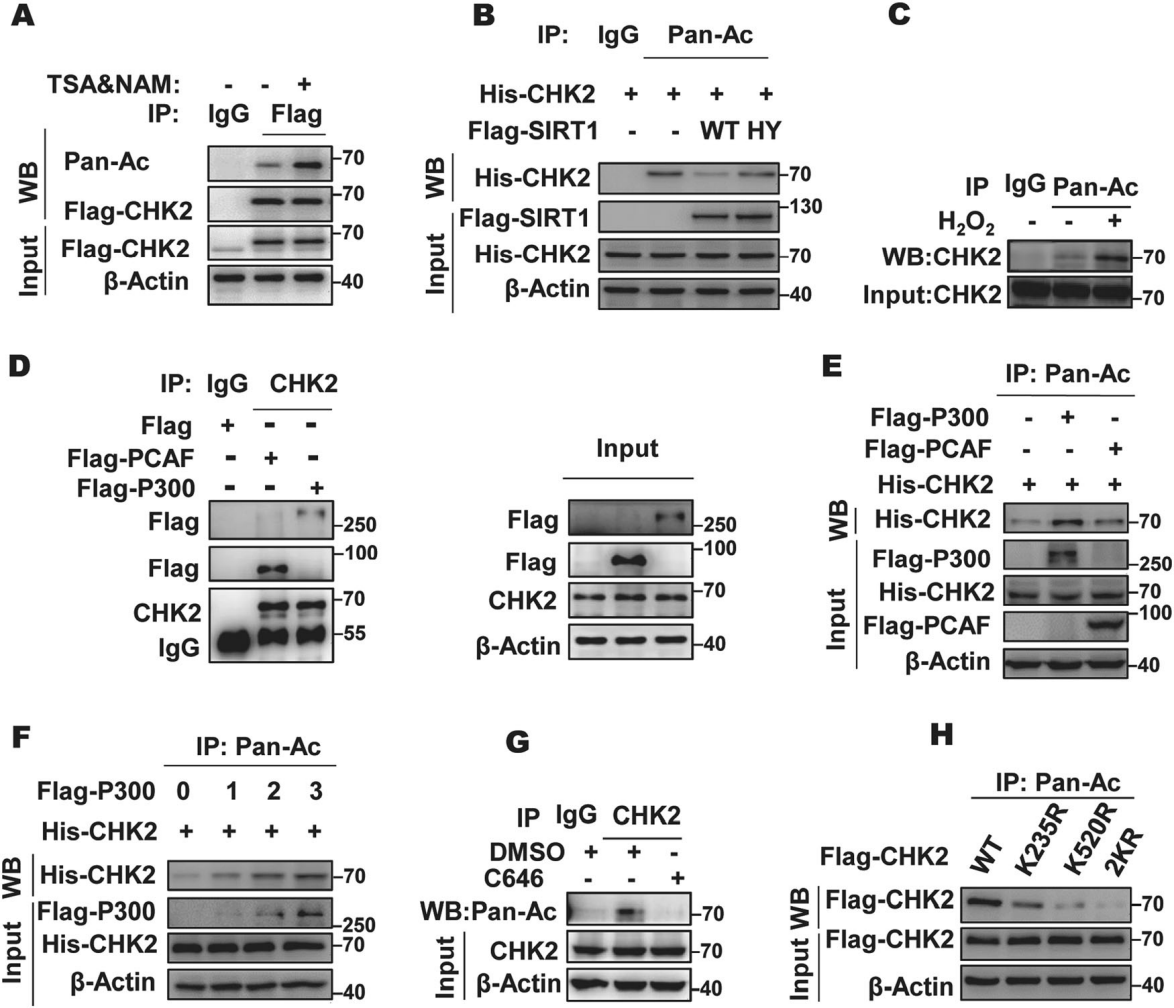
CHK2 is deacetylated by SIRT1 and acetylated by p300. **a** CHK2 is an acetylated protein. Acetylation of immunoprecipitated Flag-tagged CHK2 from HEK293 cells treated with or without histone deacetylase (HDAC) inhibitors, 5 mM nicotinamide (NAM) and 1 mM trichostatin A (TSA), simultaneously for 6 h was examined. **b** Catalytic activity of SIRT1 is required for CHK2 deacetylation. HEK293 cells expressing His-tagged CHK2 were co-transfected into Flag-tagged SIRT1 wild-type (WT) or catalytically inactive mutant H363Y. CHK2 acetylation was detected by immunoprecipitation using an anti-acetylated lysine antibody. **c** CHK2 acetylation increased under oxidative stress. HEK293 cells treated with or without 100 μM hydrogen peroxide (H_2_O_2_) for 1 h. Endogenous CHK2 acetylation was examined by immunoprecipitation (IP) and western blot. **d** Overexpressed histone acetyltransferases (HATs) p300 and p300/CBP-associated factor (PCAF) bind with CHK2. Flag-tagged p300 or PCAF was individually transfected into HEK293 cells. The interaction was detected by IP and western blot. **e** Overexpression of p300, but not PCAF, could significantly increase CHK2 acetylation. Acetylated His-tagged CHK2 was purified from cells co-transfected with His-tagged CHK2 and Flag-tagged p300 and PCAF. Acetylated CHK2 was determined by immunoblotting. **f** Different doses of recombinant human p300 were transfected into HEK293 cells stably expressing His-tagged CHK2. Acetylated CHK2 was determined by immunoblotting. **g** HEK293 cells treated overnight with dimethyl sulfoxide (DMSO) or p300 inhibitor (C646, 10 mM), or acetylated CHK2 was determined by immunoblotting. **h** HEK293 cells transfected with the indicated constructs were pre-treated with 0.5 μM EX527 for 6 h. CHK2 acetylation was examined. See also Fig. S2

Given that SIRT1 is a well-known histone and protein deacetylase, we suspected that CHK2 might be a potential SIRT1 target protein. To assess the acetylation status of CHK2, HEK293 cells were transiently transfected with Flag-CHK2, treated with specific inhibitors, lysed, and then assayed for acetylation of CHK2 via IP assay with an anti-Flag antibody or an anti-acetylated lysine antibody. Our results show that when cells were treated with an inhibitor of sirtuins, nicotinamide (NAM), and an inhibitor of HDAC classes I, II, and IV, trichostatin A (TSA), the level of acetylated CHK2 was significantly increased (Fig. 2a and Fig. S2A, S2B). To further elucidate which deacetylase is responsible for CHK2 deacetylation, TSA, NAM, and the selective inhibitor of SIRT1, EX527, were individually used to treat cells, respectively. We found that acetylation of CHK2 significantly increased after cells were treated with NAM or EX527, relative to control, suggesting a role for SIRT1 as a CHK2 deacetylase (Fig. S2C, S2D). We then verified that WT-SIRT1, but not a catalytically inactive SIRT1 mutant (H363Y), led to a significant decrease in CHK2 acetylation (Fig. 2b). Moreover, acetylation of CHK2 was diminished in cells ectopically expressing SIRT1 (Fig. S2E), while CHK2 acetylation increased both in SIRT1-knockdown cells and cells treated with the SIRT1 inhibitor EX527 (Fig. S2F, S2G). Furthermore, the level of acetylated CHK2 was significantly increased under oxidative stress context (Fig. 2c), consistent with results in Fig. 1e and Fig. S1C.

Protein acetylation is a reversible process controlled by HATs transferring an acetyl group from acetyl coenzyme A (acetyl-CoA) to the target lysine residue and by HDACs removing it. We used the protein–protein interaction (PPI) network (STRING database) to identify acetyltransferases KAT2B (PCAF) and P300 (E1A-binding protein, 300 kDa) as candidate HATs, which were proposed to specially interact with CHK2 (Fig. S2H). This result was verified by Co-IP assay (Fig. 2d). Furthermore, the ectopic expression of P300, but not PCAF, could significantly enhance the acetylation of CHK2 (Fig. 2e and Fig. S2I). In addition, a progressive increase of P300 expression resulted in increasing levels of CHK2 acetylation, while acetylation of endogenous CHK2 was abolished by treatment with the p300 inhibitor C646 (Fig. 2f, g).

To determine the acetylation sites of CHK2, two independent bioinformatical software algorithms (AESB [http://bioinfo.bjmu.edu.cn/huac/] and PHOSIDA [www.phosida.com/]) were utilized and five potential sites (K177, K214, K235, K249, K520) were identified (Fig. S3A). We mutated each individual lysine (K) to arginine (R), thereby identifying the evolutionarily conserved K235 and K520 sites as the major acetylation sites of CHK2 (Fig. S3B). While the K235R and K520R mutation resulted in an obvious reduction of CHK2 acetylation (Fig. S3C), the combined mutation (2KR) was even more potent in reducing CHK2 acetylation levels (Fig. 2h). It is well accepted that the CHK2-mediated pathway acts on p53 acetylation. To this end, we also tested the role of p300-mediated acetylation of CHK2 in p53 acetylation. However, there was no significant effect on the level of p53 (Lys382) acetylation and p53 (Ser20) phosphorylation by p300-mediated CHK2 acetylation (Fig. S3D). Furthermore, we examined subcellular localization of the non-acetylation- and acetylation-mimicking mutants of CHK2. Neither mutation affected the subcellular localization (Fig. S3E). These results suggest that p300 and SIRT1 are involved in the regulation of CHK2 acetylation.

### Acetylation at K520 promotes CHK2 activation

**Fig. 3:**
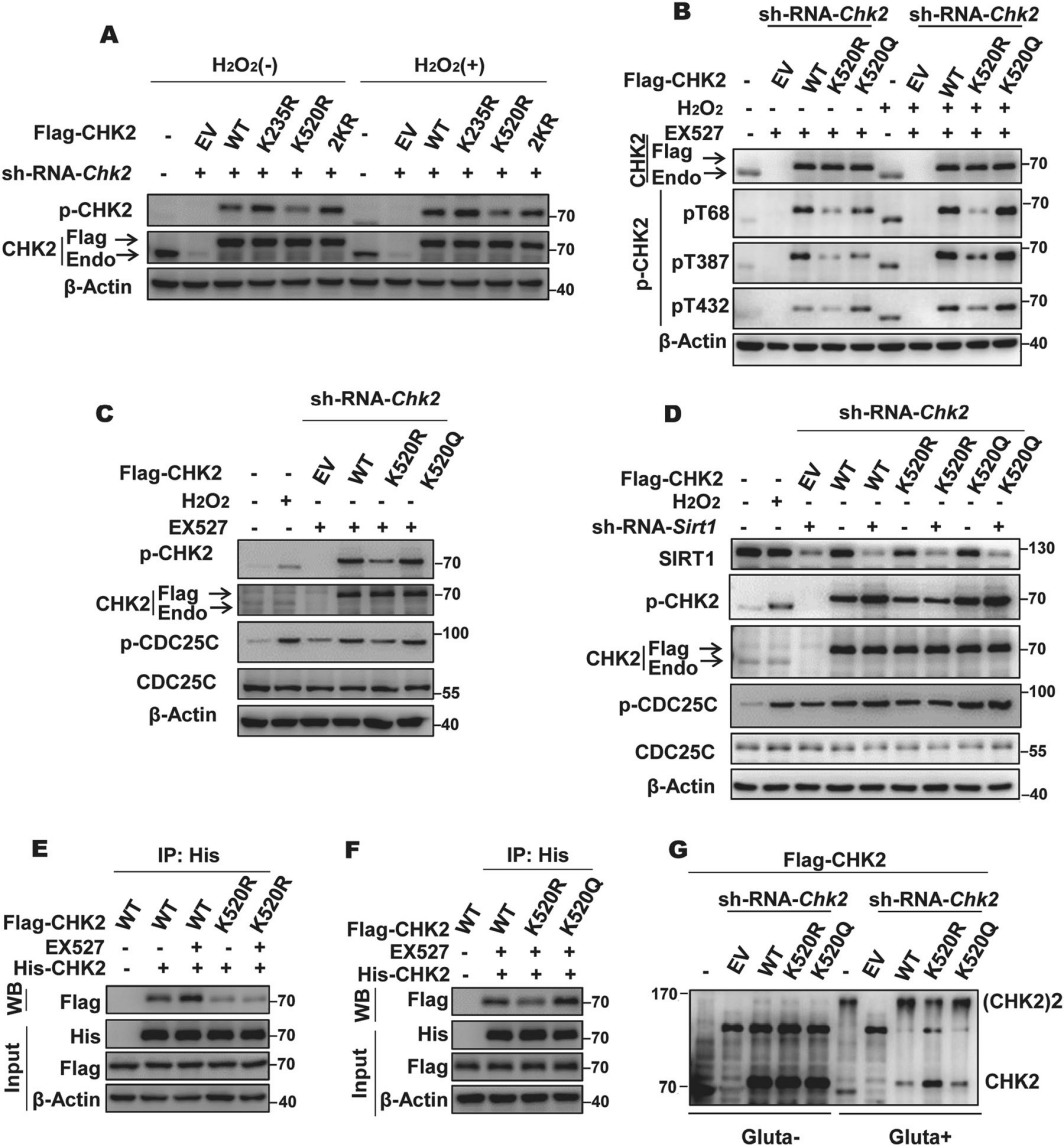
Acetylation at K520 enhances CHK2 activation. Lysine residue 520 KR mutant of CHK2 decreased the level of p-CHK2 (CHK2 phosphorylation on Threonine 68 site). **a** CHK2 phosphorylation was examined at site 68 in HCT116 cells stably expressing control or *Chk2* short hairpin RNA (shRNA) and transfected with indicated constructs. **b** CHK2 phosphorylation was examined at sites 68, 387, and 432 in HCT116 cells stably expressing control or *Chk2* shRNA and transfected with indicated constructs. **c** Deacetylation of CHK2 at K520 blocks its activity. Western blot analysis of CHK2 activation in HCT116 cells stably expressing control and *Chk2* shRNA transfected with indicated constructs. **d** HCT116 cells stably expressing control or *Sirt1* shRNA were transfected with the indicated CHK2 plasmids. CHK2 activation was determined by western blot analysis. **e** CHK2 acetylation promotes dimerization. HEK293 cells co-transfected with Flag-CHK2 (WT and K520R) and His-CHK2 vectors were exposed to 0.5 μM EX527 for 6 h or not. Cell lysates were immunoprecipitated with anti-His antibody, followed by western blot analysis with antibodies against CHK2 (Flag). **f** HEK293 cells co-transfected with indicated Flag-CHK2 plasmids and His-CHK2 vectors.CHK2 dimerization was determined as **e**. **g** HCT116 cells stably expressing control or Chk2 shRNA and reintroduced with indicated constructs were harvested and treated with or without 2.3% (v/v) glutaraldehyde (Gluta), followed by western blot analysis with antibody against CHK2. See also Fig. S3

To further understand the effect of acetylation on CHK2, we examined CHK2 activation in HCT116 cells depleted of endogenous CHK2 that were reconstituted with acetylation-deficient single mutant constructs. Interestingly, expression of the K520R construct reduced p-CHK2 levels under basal and oxidative stress conditions, while expression of the K235R had little effect (Fig. 3a and Fig. S3F). Moreover, while the expression of the K520R construct significantly diminished CHK2 autophosphorylation at T387 and T432 sites following T68 phosphorylation and its activity towards CDC25C, expression of K520Q, a mutation that mimics CHK2 acetylation, restored CHK2 activity towards CDC25C (Fig. 3b, c). Consistent results were obtained in similar reconstituted cells with or without SIRT1 knockdown (Fig. 3d). Given that CHK2 dimerization is the initial and essential step of CHK2 activation [27, 28], we next detected the dimerization status of CHK2 in cells co-transfected with His-CHK2 and Flag-WT CHK2 or Flag-K520R CHK2. Results indicated that Flag-WT CHK2-reconstructed cells displayed an increase in dimer formation in the presence of EX527, whereas the KR mutation led to an apparent decrease in dimerization (Fig. 3e). As expected, the KQ mutation increased the interaction between CHK2 monomers (Fig. 3f). A similar result was obtained from crosslinking analysis (Fig. 3g). Collectively, these results suggest that acetylation at K520 site positively regulates CHK2 activation.

### SIRT1 deficiency leads to cell cycle defects

**Fig. 4:**
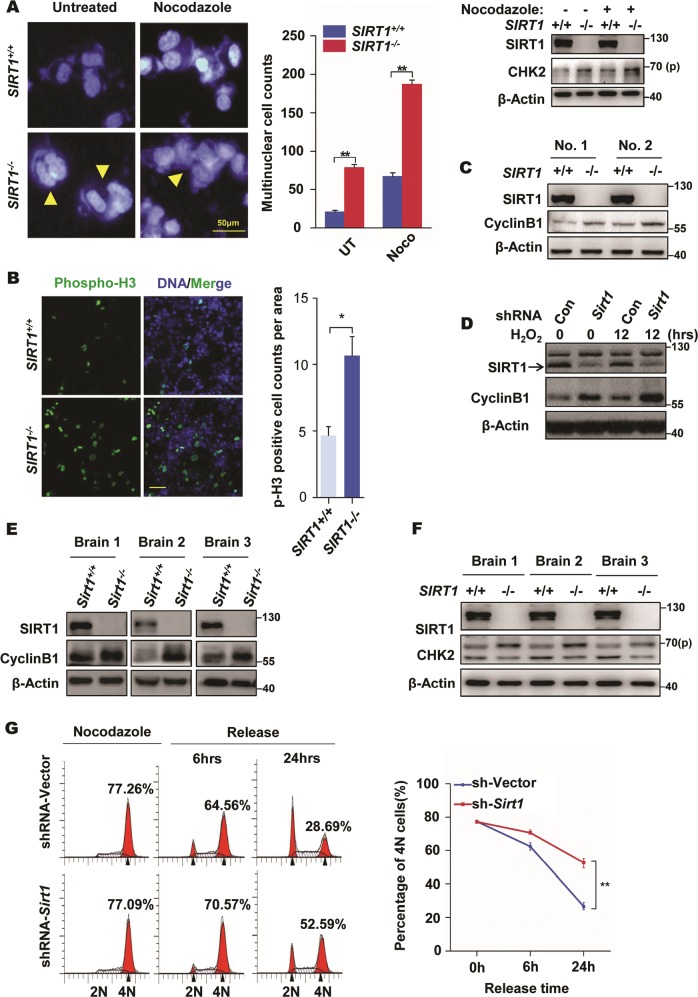
Cell cycle response to SIRT1 deficiency. **a** Morphology of *SIRT1*^*+/+*^ and *SIRT1*^*−/−*^ cells either with or without 100 ng/ml nocodazole treatment for 12 h; 4′,6-diamidino-2-phenylindole (DAPI) staining (blue) was used for morphological examination. The arrowhead indicates cells with characteristic nuclear morphologies of mitotic catastrophe. Histogram shows the counts of multinuclear cells, and data of three independent experiments were presented, mean ± SD (*n* = 300 cells). **P < 0.01 (Student’s *t* test). Meanwhile, CHK2 phosphorylation at site 68 was checked by Western blot analysis. **b** *SIRT1*^*+/+*^ and *SIRT1*^*−/−*^ mouse embryonic fibroblasts (MEFs) were stained with p-histone H3 (p-H3) antibody, and the number of p-H3-positive cells per 10,000 μm^2^ were quantified. *P < 0.05. Scale bar, 50 μm. **c–e** Western blot analysis shows the absence of the SIRT1 protein and the alterations in cyclin B1 levels in the *SIRT1*^*−/−*^ MEF cells (**c**), stably knocking down SIRT1 cells (**d**), and brains of *Sirt1*^*−/−*^ mice (**e**). **f** CHK2 phosphorylation at site 68 was examined in brains of *Sirt1*^*+/+*^ and *Sirt1*^*−/−*^ mice. **g** HELA cells stably expressing control or *Sirt1* shRNA were synchronized by nocodazole and released at the indicated times. Cells were then analyzed by fluorescence-activated cell sorting (FACS). DNA was stained with propidium iodide (PI). Results from three independent experiments are presented as mean ± SD. **P < 0.01. See also Fig. S4

A previous study has reported that the majority of *SIRT1*-null mice failed to survive postnatally [21]. However, the underlying mechanisms that contribute to the severe effect still remains elusive. As shown in Fig. S4A, we noticed a whole-body developmental retardation in perinatal *SIRT1*-null mice. To probe the phenotype, 4′,6-diamidino-2-phenylindole (DAPI) staining was done, which showed that *SIRT1*^*−/−*^ MEFs developed more multinucleated cells after nocodazole treatment and during normal culture conditions when compared with *SIRT1*^*+/+*^ MEFs, and also correlated with elevated p-CHK2 expression (Fig. 4a). Consistent with the essential role of SIRT1 in genome surveillance, we found that SIRT1 loss leads to genomic instability [22]. Furthermore, we identified that stable knockdown of SIRT1 results in significantly increased cell size compared to the control group after treatment with nocodazole, a microtubule-depolymerizing agent (Fig. S4B). According to previous reports and our findings, we suspected that *SIRT1*^*−/−*^ cells displayed the morphological phenotype of mitotic catastrophe, which is a mitotic cell death pattern distinct from apoptosis, necrosis, or senescence, and is considered as a desirable end point in cancer therapy [29–31]. To test this possibility, we examined mitotic progression in cells expressing a fusion protein of histone H2B and GFP (H2B-GFP) using time-lapse imaging. While the duration from nuclear envelope breakdown to the anaphase onset was markedly prolonged in SIRT1-knockdown cells, the increase in the frequency of cells with a hallmark of mitotic catastrophe called lagging chromosomes, which is consistent with previous studies (Figure S4C, S4D) [22, 25], showed an increased mitotic index in cells from *SIRT1*^*−/−*^ MEF (Fig. 4b). Furthermore, *SIRT1*^*−/−*^ MEF cells and stably knocking down SIRT1 cells showed higher cyclin B1 expression than WT counterparts (Fig. 4c, d), which contributed to mitotic catastrophe [32]. At the tissue level, *SIRT1*^*−/−*^ brains also increased the expression of cyclin B1 and p-CHK2 (Fig. 4e, f). Therefore, we performed Western blotting analysis and found that EX527-treated cells showed ectopic overexpression of mitotic markers of phospho-histone H3 and cyclin B1 (Fig. S4E). Moreover, SIRT1-knockout cells markedly altered the cell cycle profile with induction of a 4*N* peak, and nocodazole treatment facilitated this phenomenon (Fig. S4F). Expectedly, SIRT1 deficiency impaired the ability of cell recovery from a nocodazole-induced mitotic arrest (Fig. 4g). Altogether, these findings suggest that SIRT1 deficiency impairs normal cell cycle progression.

### CHK2 involves in SIRT1 deficiency-induced cell defects

**Fig. 5:**
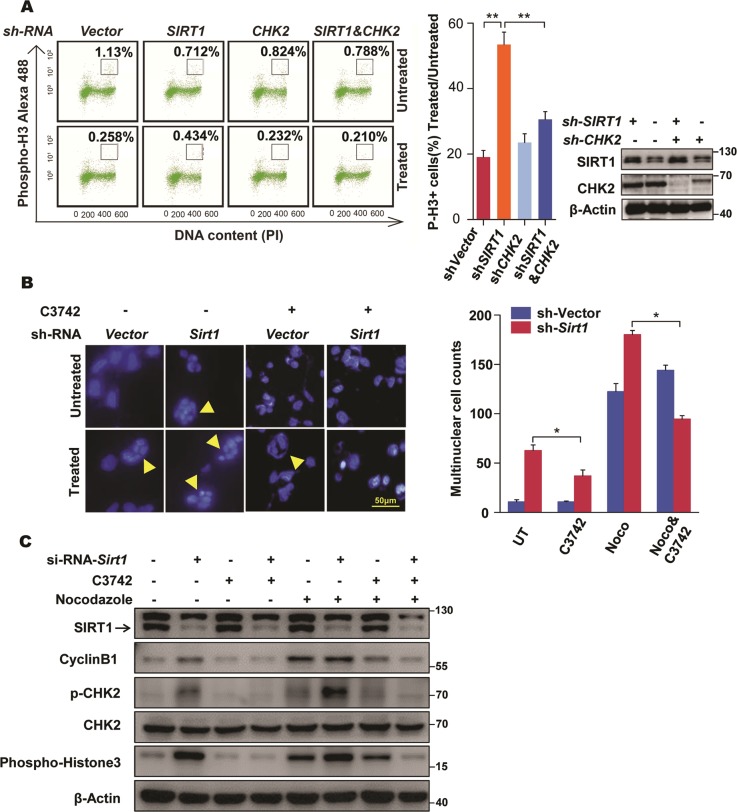
CHK2 is a master regulator of SIRT1 deficiency-induced impairments on cell. **a** Fluorescence-activated cell sorting (FACS) analysis of mitotic index in HELA cells stably expressing control, *Sirt1* short hairpin RNA (shRNA), *Chk2* shRNA, *Chk2* shRNA, and *Sirt1* shRNA treated with or without 100 μM hydrogen peroxide (H_2_O_2_) for 1 h. Statistical differences were analyzed using Student’s *t* tests. Error bars represent ± SD. **P < 0.01. Protein expression was examined by western blot with indicated antibody (right). **b** Morphological analysis of HELA cells stably expressing control and *Sirt1* shRNA either untreated or treated with 100 ng/ml nocodazole for 12 h and 10 μM of CHK2 inhibitor for 6 h as indicated. Histogram shows the percentage of multinuclear cells. Data were compiled from three independent experiments, mean ± SD (*n* = 300 cells). *P < 0.05. **c** HELA cells transiently expressing control or *Sirt1* small interfering RNA (siRNA) were treated without or with 10 μM of CHK2 inhibitor for 6 h. Cells were then incubated with nocodazole 100 ng/ml for 1 h. Mitotic markers cyclin B1 and p-H3 were measured by Western blot. See also Fig. S5

Previous studies have demonstrated that CHK2 hyperactivation (phosphorylation), during mitosis, is responsible for increasing the frequency of lagging chromosomes [33, 34]. We then sought to address the functional significance of dysregulated activation of CHK2 in cell response to SIRT1 depletion. The expression of p-CHK2 revealed an association with lagging chromosome in SIRT1-deficient cells, consistent with a previous study (Fig. S5A) [34]. In addition, cell cycle profile in HCT116 cells under EX527 treatment conditions with or without additional selective CHK2 inhibitor (C3742) were examined. As shown in Fig. S5B, inhibition of CHK2 activity represses the high 4*N* peak induced by SIRT1 inhibition. Fluorescence-activated cell sorting (FACS) analysis indicated that the increased phospho-histone H3 levels induced by oxidative stress treatment in SIRT1-deficient cells were attenuated by simultaneous CHK2 abrogation (Fig. 5a), indicating that SIRT1 collaborates with CHK2 to regulate the mitotic stress response. Morphologically, inhibiting CHK2 activity also reversed the phenomenon of multinucleation induced by SIRT1 depletion (Fig. 5b). Further evidences were observed in MEF cells by the downregulation of CHK2 expression (Fig. S5C–5E). Moreover, CHK2 inactivation limited the increased levels of phospho-histone H3 and cyclin B1 caused by SIRT1 depletion with a specific small interfering RNA (siRNA) (Fig. 5c). Taken together, these results imply that CHK2 may be an essential component of the cell cycle defects seen in SIRT1-deficient cells.

### CHK2 is required for SIRT1 deficiency-driven neonatal lethality

**Fig. 6:**
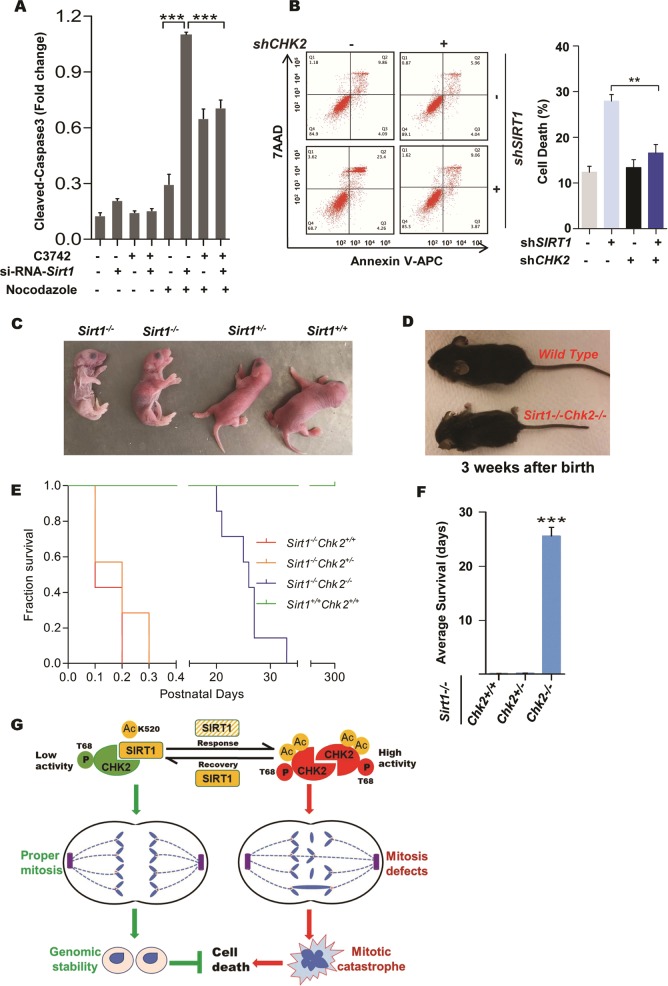
CHK2 deficiency promotes survival upon the absence of SIRT1. **a** HELA cells transfected with the control and *Sirt1* small interfering RNA (siRNA) were treated with or without 10 μM of C3742 incubated in media containing nocodazole 50 ng/ml for 24 h or not. Cell lysates were blotted and results from three independent experiments were presented as the histogram. ***P < 0.001. **b** HELA cells with indicated genotypes were incubated in medium containing nocodazole 100 ng/ml for 24 h. Representative FACS analysis of apoptosis. Results from three independent experiments are presented as the histogram. **P < 0.01. **c** Photograph of 24-h pups after birth with indicated *Sirt1* status. **d** Photograph of post-natal 3-week-old wild-type, *Sirt1*^*−/−*^*Chk2*^*−/−*^ mice. **e** Kaplan–Meier survival curves of indicated animals (*n* ≥ 7 mice/genotype). **f** Average lifespan of consecutive births of *Sirt1*^*−/−*^ pups with the indicated *Chk2* status. **g** Schematic model we proposed for the role of SIRT1–CHK2 axis in cell cycle control. See also Fig. S6

Frequently, there are two ultimate fate of cells undergoing prolonged mitotic arrest—they can either die (often apoptosis) in mitosis or slip out of aberrant mitosis (with unrepaired damage) and die in the following cell cycles [29]. Since SIRT1 loss leads to apoptosis [35, 36], it remains unknown whether it is involved in cell death following mitotic catastrophe. As such, we performed western blotting analysis, and the results showed that SIRT1 depletion increased the level of cell death following sustained mitosis arrest (Fig. S6A). As expected, treatment of cells with C3742 suppressed cell death following nocodazole-induced mitotic catastrophe both in SIRT1-knockdown cells and in SIRT1 deacetylase-inactivated cells (Fig. 6a and Fig. S6B, S6C). FACS analysis showed similar results (Fig. 6b). These results are consistent with a previous study [37]. As shown in Fig. 6c, mice lacking the gene *Sirt1* die a few hours after birth. Finally, to extend our observations to a whole-organism level, we assessed the effects of *Chk2* deletion on *Sirt1*-knockout animals. We observed that *Chk2* deficiency resulted in neonatal lethality of *Sirt1*-knockout mice (with a deletion of exon 4) and extended the survival time from 2 to 4 h (0.1–0.2 days) to adulthood (average of 25.6 days) (Fig. 6d–f). Similar analysis using another Sirt1 mutant mice with a deletion of exons 5 and 6 also revealed that the absence of Chk2 allows these *Sirt1*-null mice to escape from post-natal lethality (Fig. S6D). Taken together, these results suggest that the SIRT1 deficiency-driven defects in cells and organisms, at least in part, are attributed to CHK2.

## Discussion

In this present work, we reveal that acetylation/deacetylation modulates the function of CHK2 in cell cycle by controlling its activation. Conventionally, SIRT1 inactivates CHK2 by directly binding to and deacetylating CHK2, which in turn suppresses its phosphorylation on the T68 site, thus maintaining its low activity and allowing proper mitosis. In the absence of SIRT1, hyperacetylated CHK2 promotes its hyperphosphorylation and dimerization, thus leading to inappropriate activation of CHK2, an event that drives mitosis defects and subsequent cell death via mitotic catastrophe (Fig. 6g). Acetylation of CHK2 therefore provides a previously unknown regulatory form of CHK2 activation, revealing a mechanism in maintaining genomic stability.

Many functions of SIRT1 have been disclosed since its discovery. Previous studies have demonstrated that SIRT1 mutant cells displayed p53 hyperacetylation upon DNA damage and increased IR-induced apoptosis in thymocytes [21]. Recently, it has been reported that SIRT1 depletion induces chromosome aneuploidy by modulating spindle dynamics through Plk1 activity regulation [25]. Our work suggests that SIRT1 deficiency results in pronounced multinuclear morphology, which further leads to mitotic catastrophe (Fig. 4a–c). Moreover, CHK2 inactivation could mitigate a number of effects of SIRT1 deficiency-induced impairments on cellular homeostasis. On the other hand, the absence of SIRT1 leads to impaired DNA damage repair and DNA replication fork initiation [22, 38]. These lines of evidence together support SIRT1 is an indispensable stress-response protein. Nevertheless, further studies are required to exhaustively determine the underlying molecular mechanisms by which SIRT1 is involved in governing genome integrity.

Emerging evidence suggests that genomic instability is a master cause of aging and cancer. To maintain the precise replication of the genome and ensure the continuous surveillance of its integrity, cells have evolved various response mechanisms. The DDR is an essential modulator of cellular homeostasis and is involved in various diseases, including aging and cancer [39, 40]. In particular, DDR functions as a critical mechanism of balancing the scale between cancer and aging [40]. Previous studies indicate that CHK2 serves as a well-known transducer kinase for the DDR, and senses serine/threonine kinase ATM activity via a phosphorylation cascade, to coordinate cell cycle progression with DNA repair and cell survival or death [41]. Recently, a negative correlation between CHK2 and cell and tissue turnover has been reported [42]. In addition, CHK2 germline mutations and rare somatic mutations have been detected with high incidence in a number of familial cancers in humans, including prostate, breast, ovarian, thyroid, kidney, colorectal and bladder cancers, and leukemias [43, 44], suggesting that CHK2 may be a good target in cancer therapy.

The interplay of different PTMs has been regarded as a key mechanism for the cellular signaling dynamic control [45]. CHK2 has previously been shown to be subject to phosphorylation and ubiquitination [46, 47]. Phosphorylation of CHK2 has been the best studied PTM, while relatively little is known about how other modifications are integrated into CHK2 signaling. Interestingly, evidence suggests that acetylation of ATM regulates its kinase activity [48]. Here, we show CHK2 is also regulated by acetylation and that acetylation affects CHK2 dimer formation, a prerequisite of its molecular activation (Fig. 3e–g). Unlike CHK2 activation, many aspects of its inactivation remain poorly explored. Here we provided a framework of CHK2 deactivation by its modification of acetylation. Possibly, the structural analysis of acetylated CHK2 could help us to better understand the atlas. It is conceivable that alternative regulatory pathways of SIRT1-mediated CHK2 inactivation may exist. In that regard, further studies are necessary to reconcile if this regulation may also be caused by its kinase (ATM, DNA-PKcs) or phosphatase (PP2A, WIP1, PP1) changes.

The question is: the phenotypes in mice vary from genetic background and targeting strategy of *Sirt1* gene. In this regard, it is worth noting that the mice we employed in our study were those carrying a deletion of exon 4 with a 129SVEV/B6C57 background. Studies have shown that about 90% of these mutant mice exhibited perinatal lethality [21]. Additionally, those mutant mice generated by using TC1 ES cells like us, but with a deletion of exons 5 and 6, showed more severe phenotype both in the 129SVEV/FVB background and in the 129SEVE/FVB/Black Swiss background [22]. However, McBurney’s mice with *Sirt1* mutant strain of replacing exons 5 and 6 with a hygromycin gene were generated by using R1 ES cells and showed minimal perinatal lethality either in 129SV-CP background or in 129/CD1 mixed background [49]. Of particular interest, another evidence from mice containing the deletion of exons 5 and 6 (shown in Fig. S6D) showed that *Chk2*^*+/−*^ displayed a complete rescue of *Sirt1* absence, whereas *Chk2*^*−/−*^ displayed partial rescue. This implies that the function of *Chk2* varies in different *Sirt1* deletion context. However, the CHK2 protein impact on the functionality of SIRT1 at the whole organism remains to be further investigated. Our findings of concomitant *Chk2* depletion rescues the neonatal lethality of *Sirt1*-loss mice, and places CHK2 in a critical position in SIRT1 pro-survival signaling.

In summary, these results provide one possible explanation for how SIRT1 dysregulation leads to genomic instability and ultimately aging or cancer progression. It will therefore be of interest to fully define the importance of CHK2 in multiple SIRT1-orchestrated signaling pathways.

## Materials and methods

### Cell culture

Three human tumor cell lines and one mouse-derived cell line were used: H1299, HELA, HCT116, and 293T were obtained from cell bank of Cao’s lab. *Sirt1*^*+/+*^ and *Sirt1*^*−/−*^ MEFs were established from *Sirt1*^*+/−*^-mated E10.5–15.5 embryos (with a deletion of exon 4). HCT116 and H1299 cells were cultured in RPMI-1640 medium supplemented with 10% fetal bovine serum (FBS), 293T, and HELA in Dulbecco’s modified Eagle’s medium (DMEM) supplemented with 10% FBS, and MEFs were maintained in DMEM containing 15% FBS.

### Chemoradiation

A biological x-ray irradiator RS2000pro (Rad Source Technologies, USA) was used to irradiate the cells at a dose rate of 4.125 Gy/min with a radiation output of 160 kV at 25 mA.

### Mice

*Sirt1*^*+/−*^ mice with a deletion of exon 4 were a kind gift from Cheng et al. [21]. *Chk2*^*+/−*^ mice were a kind gift from Takai et al. [50]. These two genotypes of mice were crossed to generate double-mutant mice. Animal use and care protocols, including all operation procedures, were approved by the Animal Care and Use Committee of China Medical University.

### FACS analysis

For analysis of apoptosis, 2.5 × 10^5^–2 × 10^6^ cells were plated into a 60 mm dish. Twenty-four hours after plating, cells were treated with nocodazole or dimethyl sulfoxide (DMSO) as a control. Cells were harvested with EDTA-free trypsin and then stained with an anti-Annexin V FITC (BD Biosciences) or anti-APC (Keygen, China) antibody. Alexa Fluor 488 goat anti-mouse IgG (HtL) (Molecular Probes) was used for the secondary antibody. Nuclei were labeled with propidium iodide (PI) or 7-amino-actinomycin D for fluorescent labeling. Cells were analyzed by FACS caliber (Becton Dickinson). To analyze cell cycle arrest, cells were treated with H_2_O_2_ with 200 μM/L or phosphate-buffered saline (PBS) as a control. After trypsinization, cells were fixed by 70% ethanol in −20 °C icebox overnight and then treated with 2 N HCl and 0.5% Triton X-100. We used an anti-phospho-histone H3 (Ser10) (Cell Signal Technology) antibody followed by an Alexa Fluor 488 goat anti-rabbit IgG (HtL) (Molecular Probes) as a second antibody with PI. Cells were analyzed by FACS caliber (Becton Dickinson).

### Western blot analysis and immunoprecipitation

Western blot analysis was performed using standard procedures for whole-cell extracts from cell lines. Antibodies used include Chk2 (1:5000, Becton Dickinson and Millipore), SIRT1 (1:2000, Millipore), acetyl-lysine (1:1000), phospho-CHK2-T68 (1:1000), phospho-CHK2-Thr387 (1:1000), phospho-p53-Ser20 (1:1000), acetyl-p53-Thr382 (1:1000), phospho-histone H2A.X (Ser139) (1:1000), phospho-ATM-Ser1981 (1:1000), ATM (1:1000), phospho-histone H3-S10 (1:1000), p-CDC25C (ser216) (1:1000), CDC25C (1:1000), cleaved PARP-1 (1:1000) and cleaved caspase-3 (1:1000) (Cell Signaling Technology), phospho-CHK2-Thr432 (1:500, Invitrogen), P53 (Do-1, 1:1000, Santa Cruz Biotechnology), FLAG (clone M2) (1:2000), α-tubulin (1:5000, Sigma), and β-actin (1:5000, Sigma). For immunoprecipitation analysis, cell lysates (1–4 mg) after preclearing were mixed with antibodies (2 μg) at 4 °C overnight followed by the addition of 30 μl of protein-G (for mouse antibodies)- or protein-A (for rabbit antibodies)-coupled sepharose beads (GE) for 3 h at 4 °C. Immune complexes were washed three times with lysis buffer [50 mM Tris (pH 7.4), 1% Triton X-100, 0.5% Nonidet P-40, 150 mM NaCl, protease, phosphatase inhibitor mixture (Sigma)]. After boiling in 2× loading buffer, samples were subjected to SDS-PAGE, and then scanned using ECL.

### GST fusion protein purification and GST pull-down assays

The bacterial expression constructs (pGEX-4T-2 or 5X-1) containing the indicated genes were transformed into BL21-competent cells (Takara). Cells were induced to overexpress the protein for 3 h while shaking at 30 °C. Cells were resuspended in bacterial lysis (PBS containing 0.5% Triton X-100, 5 mM β-mercaptoethanol, 1 mM PMSF, and 2 mM EDTA), followed by ultrasonication. The proteins were purified by a single step using glutathione bead according to the manufacturer’s protocol (Promega Science). GST pull-down assays were performed as previously described [51].

### Agents, plasmids, and transfections

Nocodazole, EX527, DMSO, C3742, and C646 were all obtained from Sigma, Lipofectamine 2000 was from Invitrogen, and Higene was from APPLYGEN. Glutaraldehyde was purchased from Sangon Biotech. The Flag-tagged SIRT1 WT or deacetylase-inactive mutant (HY) and SIRT1 siRNA retrovirus have been previously described [23, 52, 53]. SIRT1 and CHK2 siRNA retrovirus were purchased from GeneChem. The method of making special gene stable knockdown cells was as described previously [53]. In the knockdown experiments, cells were transfected using Oligofectamine (Invitrogen) according to the manufacturer’s protocol. Infected cells were identified by western blotting after 36 h of RNAi transfection. Stable clones of cells stably expressing Flag-SIRT1 WT, Flag-SIRT1 HY, or the empty vector Flag we used were selected by hydromycin (1000 mg/ml, Sigma). Site-directed mutagenesis of Flag-CHK2 was performed using QuikChange XL (Stratagene) and confirmed by sequencing.

### Crosslinking assay

Glutaraldehyde crosslinking experiment was used to detect the CHK2 dimer. Briefly, 100 μl of the whole-cell lysates was incubated at 37 °C for 5 min with 5 μl of freshly prepared 2.3% (v/v) glutaraldehyde. Then, the reaction was stopped by adding 10 μl of 1 M Tris-HCl (pH 8.0) and then subjected to western blot analysis.

### Time-lapse imaging

HEK293/GFP-H2B stable cell lines were seeded on a four-chambered 35 mm dish. Images were collected every 15 min for 48 h using a ×20 lens objective on an inverted fluorescence microscope (Nikon Biostation IM-Q) equipped with an environmental control chamber. The temperature of the imaging medium was kept at 37 °C. Image sequences were viewed and the cell behaviors were analyzed manually.

### Immunofluorescence

MEF cells and stable knockdown cells were washed twice with ice-cold PBS, fixed with 4% buffered paraformaldehyde, and permeabilized with 0.5% Triton X-100 for 10 min. Cells were then incubated with 4′,6-diamidino-2-phenylindole (DAPI) at room temperature for 15 min, and then the glass slides were examined using immunofluorescence microscope (Olympus).

### Acetylation analysis

HEK293 and HCT116 cells were transfected with WT Flag-CHK2 and then treated with TSA (1 mM) and NAM (5 mM). After 6 h, treatment cells were harvested and the cell pellet was resuspended in 500 μl of lysis buffer containing 10 mM TSA and 5 mM NAM, and thus the lysate was subjected to immunoprecipitation and western blot analysis as stated above. SIRT1 inhibitor EX527 was added 4 h for further exploring SIRT1-induced deacetylation of CHK2, as well as SIRT1-controlled deacetylated residues of CHK2 after in silico prediction. Flag-CHK2 was purified by immunoprecipitation with M2 beads and eluted by 3× FLAG peptide (0.125 mg/ml). Then, 2 μg of purified Flag-CHK2 was incubated in 30 μl of reaction mixture containing 100 mM acetyl-CoA and 1 μg purified p300 protein (Enzo Life Sciences) at 30 ℃ for 1 h. The samples were then subjected to western blot analysis.

### Statistics

The statistical analysis was carried out with Student’s *t* test as indicated by using the SPSS (20.0) statistical software program (SPSS Inc., USA). Respective *P* values as a measure of significance are indicated.

## Supplementary information

Revised Supplemental results

Supplementary Video 1

Supplementary Video 2

Supplementary Video 3

## Data Availability

The data that support the findings of this study are available from the authors on reasonable request, see author contributions for specific data sets.
