# Supplementary material for: SIRT1 modulates cell cycle progression by regulating CHK2 acetylation−phosphorylation
Source: Cell Death Differ. 2019 Jun 17;27(2):482–96. doi: 10.1038/s41418-019-0369-7 (PMC7206007; doi:10.1038/s41418-019-0369-7)
Supplement: Supplementary file 1 — Revised Supplemental results [file 41418_2019_369_MOESM1_ESM.docx]

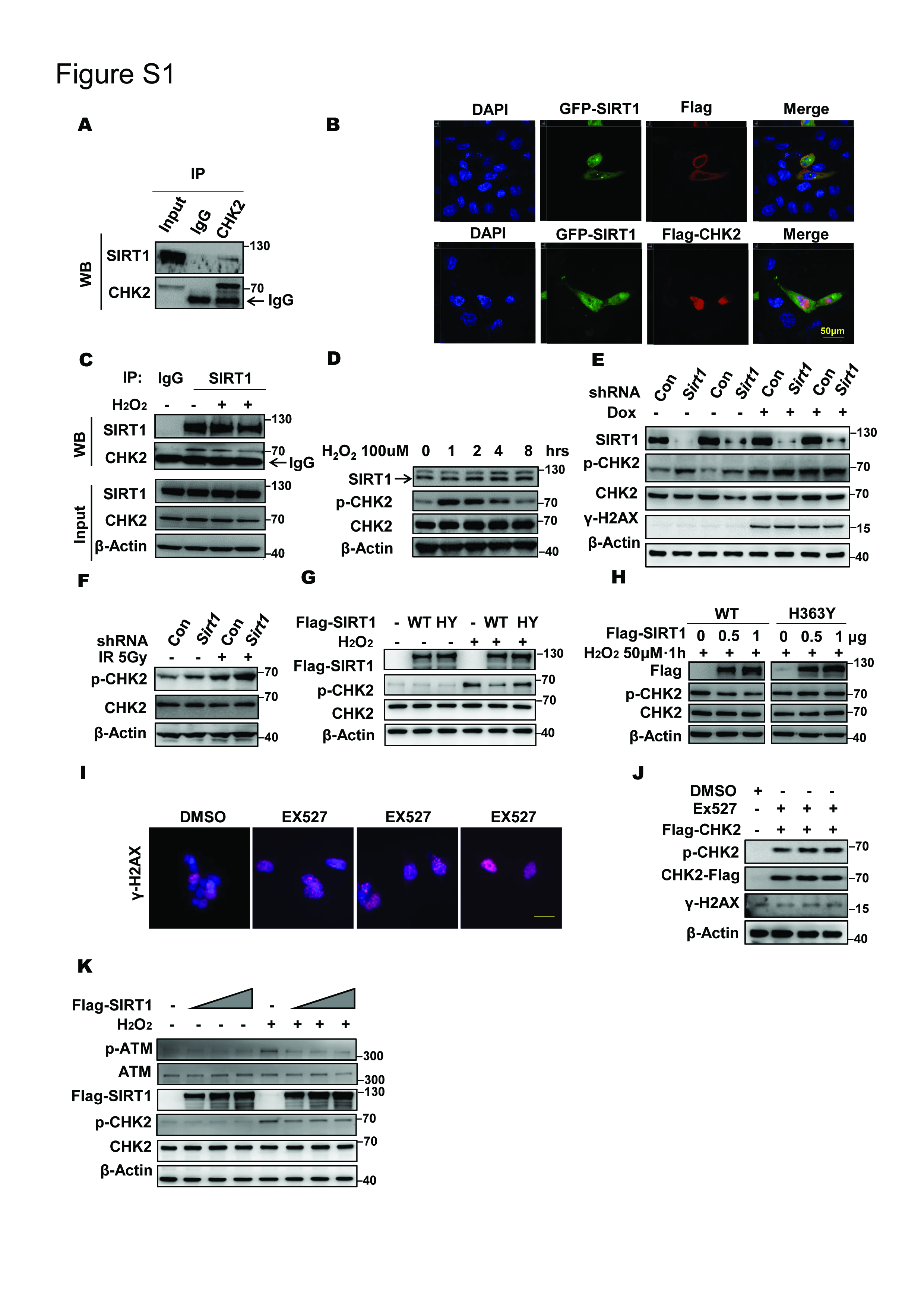


Figure S1 **SIRT1 interacts with CHK2 and** **suppresses its phosphorylation.**

(A) MEF cell lysates were subjected to immunoprecipitation with control IgG, anti-CHK2 antibodies and the immunoprecipitates were blotted with the indicated antibodies. (B) HEK293 cells were transfected with the indicated constructs. Colocalization between SIRT1 and CHK2 was determined by confocal Laser Scanning Microscope. (C) H_2_O_2_ treatment decreased the binding of SIRT1 to CHK2. Endogenous immunoprecipitation with control IgG, or anti-SIRT1 antibodies in HELA cells treated with 200μM/L or 500μM/L H_2_O_2_ for 1 hour. Immunoprecipitates were then blotted with the indicated antibodies. (D) HEK293 cells were treated with 100μM H_2_O_2_ at the indicated times, then cells were lysed and cell lysates were measured with the indicated antibodies using Western blot. (E) H1299 and HCT116 cells stably expressing control or *Sirt1* shRNA were treated with or without 200ng doxorubicin for 16 hours, followed by Western blot analysis with indicated antibodies. (F) H1299 cells stably expressing control or *Sirt1* shRNA were irradiated at 5Gy and released for 1h. Cell lysates were subjected to western blot analysis. (G) H1299 cells transfected with empty vector, FLAG-SIRT1 WT or catalytically inactive mutant H363Y were treated with or without 100μM H_2_O_2_ for 1 hour. CHK2 phosphorylation was determined by Western blot. (H) Different doses of Flag-tagged SIRT1 WT or catalytically inactive mutant H363Y were transfected into HEK293 cells. Then the cells treated with 50μM H_2_O_2_ for 1 hour. CHK2 phosphorylation was measured by Western blot. (I) Immunofluorescent staining detected no significant change in γ-H2AX after treating with or without EX527 at 0.5μM for 0h,3h,6h and 9h. (J) Flag-tagged CHK2 was forced overexpressed in HCT116 cells stably expressing *shCHK2.* Cells were treated with or without 0.1μM, 0.5μM and 1μM EX527 for 6h, followed by western blot analysis with indicated antibodies. γ-H2AX served as the DNA damage marker. (K) HCT116 cells transfected with Flag or gradual amount of Flag-SIRT1 plasmids were exposed to 100μM H_2_O_2_ for 1 hour or not. Cells were harvested and subjected to Western blot with indicated antibodies. Related to Figure 1.


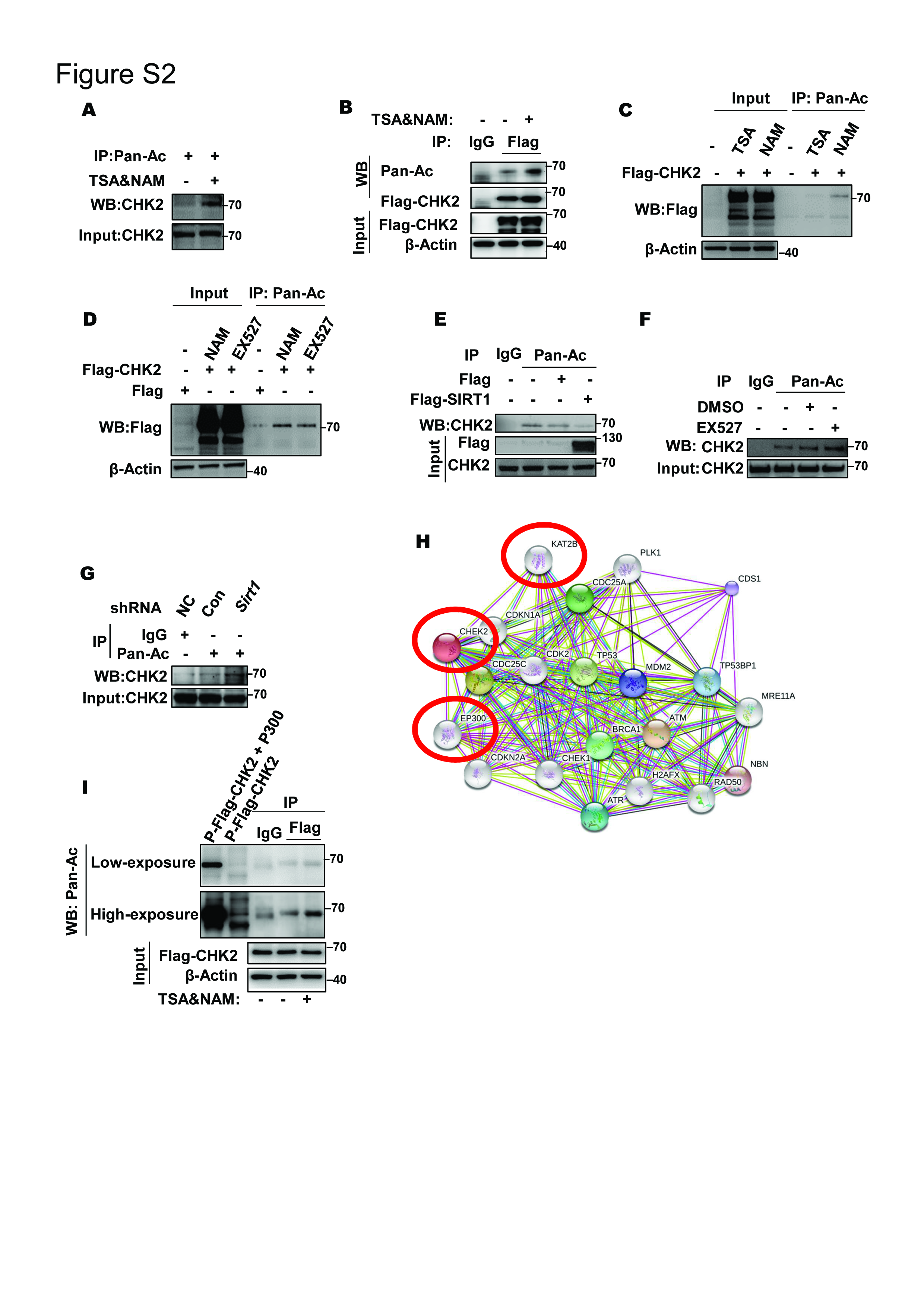


Figure S2 **p300 and SIRT1 regulate CHK2 acetylation.**

(A)Endogenous CHK2 is acetylated. Acetylated proteins were immunoprecipitated with the antibody to acetylated lysine from HEK293 cells treated with or without HDAC inhibitors TSA (1mM) and NAM (5mM) simultaneously for 6h. Acetylation of endogenous CHK2 protein was analyzed with western bolt analysis. (B) Acetylation of immunoprecipitated Flag-tagged CHK2 from HCT116 cells treated with or without HDAC inhibitors 5 mM NAM and 1 mM TSA simultaneously for 6 h was examined. (C) HEK293 cells transfected with FLAG-CHK2 were either treated with TSA(1mM) or NAM (10 mM) for 12 hours. CHK2 acetylation was determined by IP and Western blot. (D) Cells as (C) were either treated with NAM (10 mM) or EX527 (0.5μM) for 12 hours. Cell lysates were subjected to immunoprecipitation to measure CHK2 acetylation. (E) CHK2 acetylation in HEK293 cells transiently expressing control or Flag-tagged SIRT1 was determined by IP and Western blot. (F) Endogenous CHK2 acetylation was examined in HEK293 cells treated with or without EX527(0.5μM,6h). (G) CHK2 acetylation was examined in HEK293 cells stably expressing control or *Sirt1* shRNA. (H) Graphic of SIRT1-associated proteins identified by STRING Protein-Protein Interaction Networks analysis. (I) Acetylation of immunoprecipitated Flag-tagged CHK2 from HEK293 cells treated with or without HDAC inhibitors NAM and TSA simultaneously as above was examined. Acetylated Flag-CHK2 by p300 in vitro denotes positive control. Related to Figure 2.


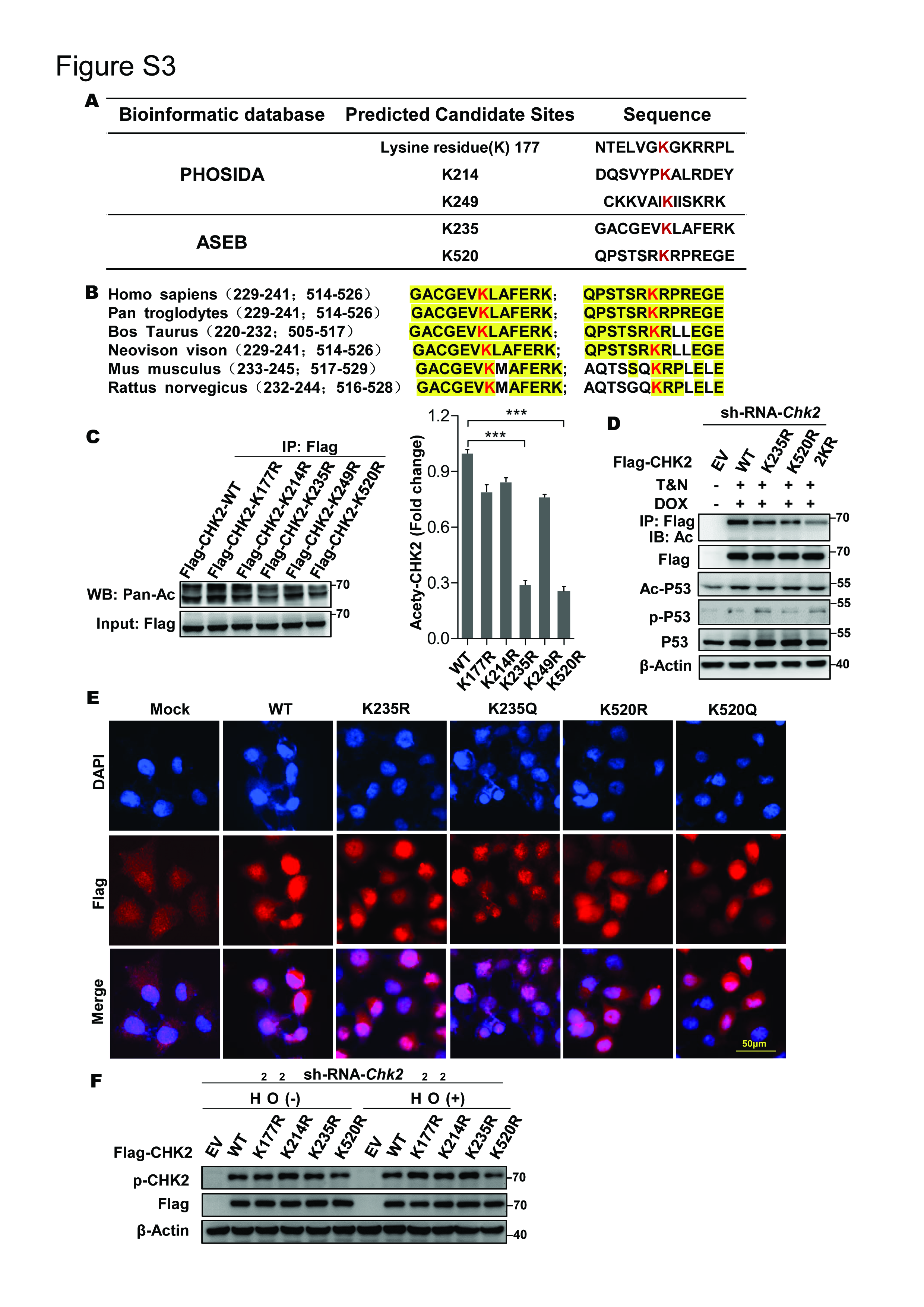


Figure S3 **Identification of CHK2 acetylation sites.**

(A) List of predicted CHK2 acetylation sites identified by computational analysis. (B) Alignment of the similarity and identity of protein sequences in different CHK2 vertebrate orthologs. The red color indicates the identified acetylated lysine residues of CHK2. (C) Acetylation of ectopically expressed WT and K177R, K214R, K235R, K249R, K520R individual mutants was detected by IP and Western blot. Data from three independent experiments were presented as the histogram. ***p < 0.001. (D) HCT116 cells stably expressing *shCHK2* were further transfected with Flag-CHK2 wild type or its mutants as indicated. Cells were treated with 200ng doxorubicin for 24 hours. Cells were harvested and subjected to IP or western blot analysis. (E) Representative immunofluorescent images of HEK293 cells stably expressing *shCHK2* reintroduced with indicated Flag-CHK2 constructs. DNA was stained with DAPI to visualize the nucleus. Scale bars, 50 μm. (F) Flag-tagged WT CHK2 and constructs with mutation of lysine residues (K177、K214、K235 and K520) to arginine were transfected into HCT116 cells stably expressing *Chk2* shRNA.CHK2 phosphorylation was examined by Western blot in absence or presence of 100μM H_2_O_2_. Related to Figure 3.


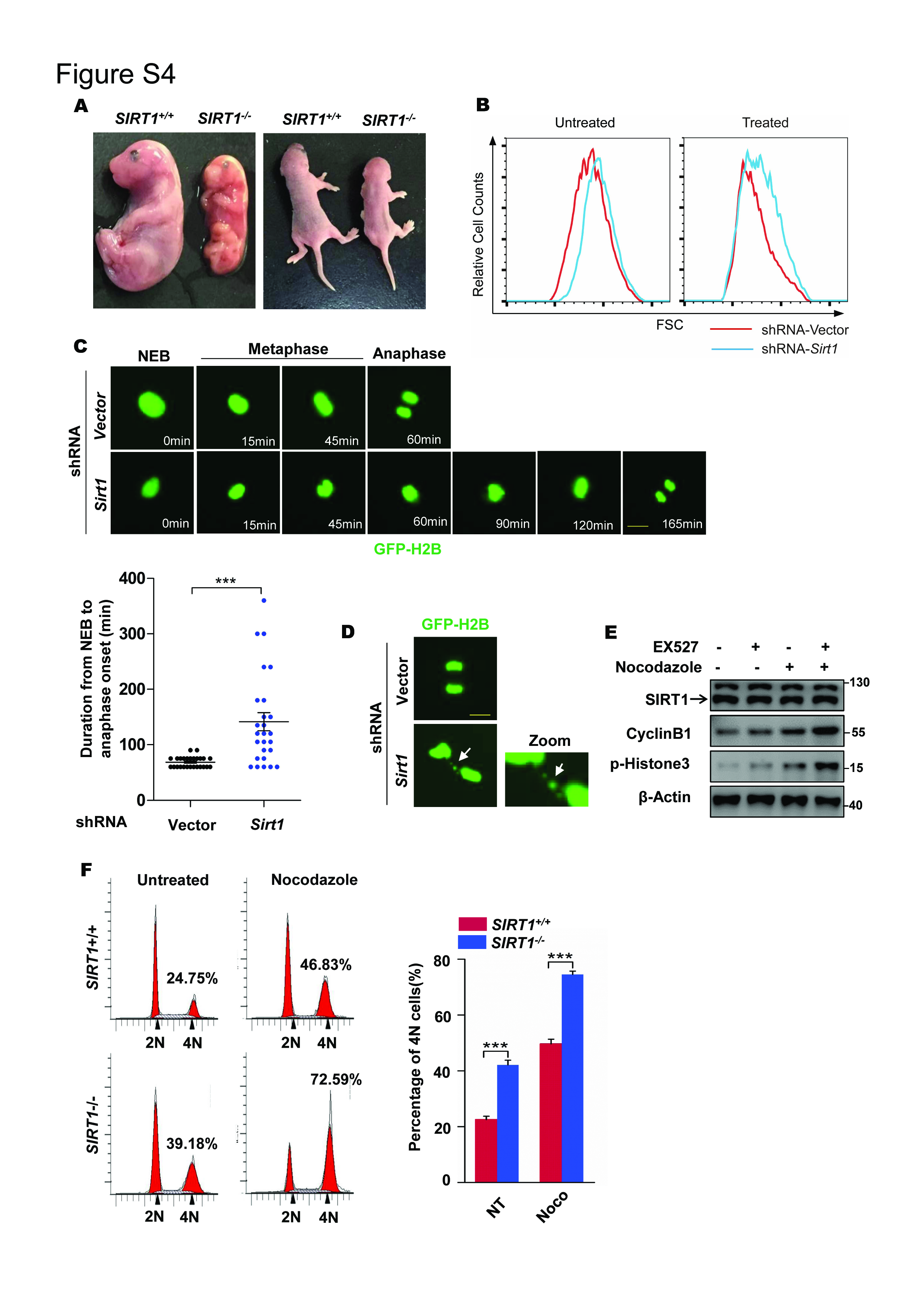


Figure S4 **Impairments of SIRT1 loss.**

(A) Photographs of E19.5 and newborn *SIRT1^+/+^* and *SIRT1^−/−^* embryos. (B) Cell size of HCT116 cells stably expressing control or *SIRT1* shRNA either treated with 100ng/ml nocodazole for 12h or not were examined by FACS analysis. (C) HEK293 cells stably expressing control shRNA or *shSIRT1* co-transfected with GFP-H2B. Mitotic progression was imaged. See also Supplementary Videos 1 and 2. Selected frames from time-lapse videos are shown. Scale bar = 50μm. The length of time from NEB to anaphase onset was analysed. n= 26. (D) Representative image of mitotic cells in HEK293 cells stably expressing control shRNA or *shSIRT1* together with ectopic H2B-GFP (arrowhead: lagging chromosome, scale bar represents 50 μm). See also Supplementary Video 3. (E) HEK293 cells were incubated with media containing 100ng/ml nocodazole or not, then treated with or without 0.5 μM of EX527 for 6 hours. Cells were harvested and lysates were blotted with the indicated antibodies. (F) *SIRT1^+/+^* and *SIRT1^−/−^* MEFs were treated with or without 100ng/ml of nocodazole and harvested after 6 hours for FACS analysis. PI-staining for DNA profile analysis. Histogram showing statistical differences from three independent experiments. ***p < 0.001. Related to Figure 4.


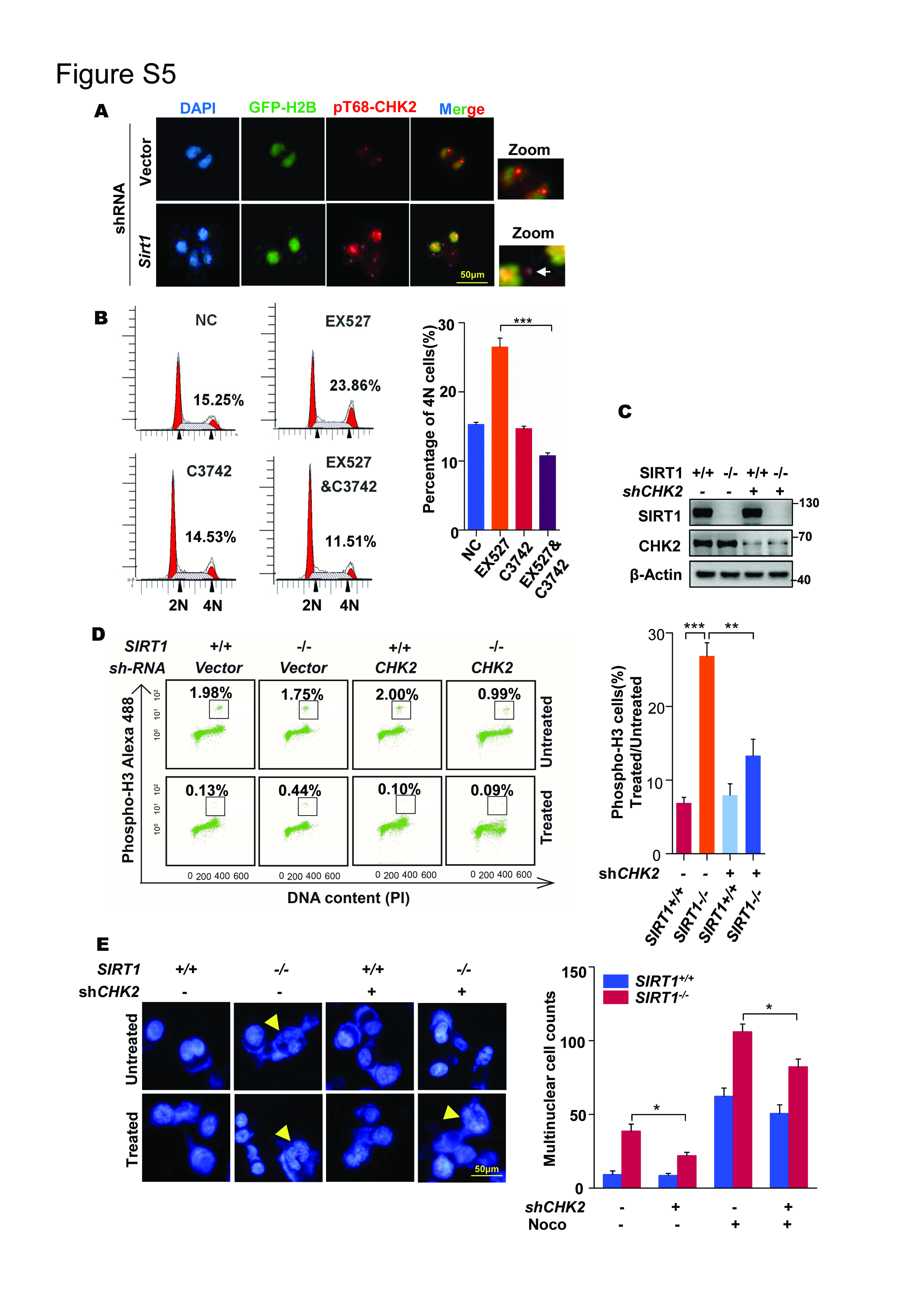


Figure S5 **CHK2 is required for SIRT1 deficiency caused impairments on cells.**


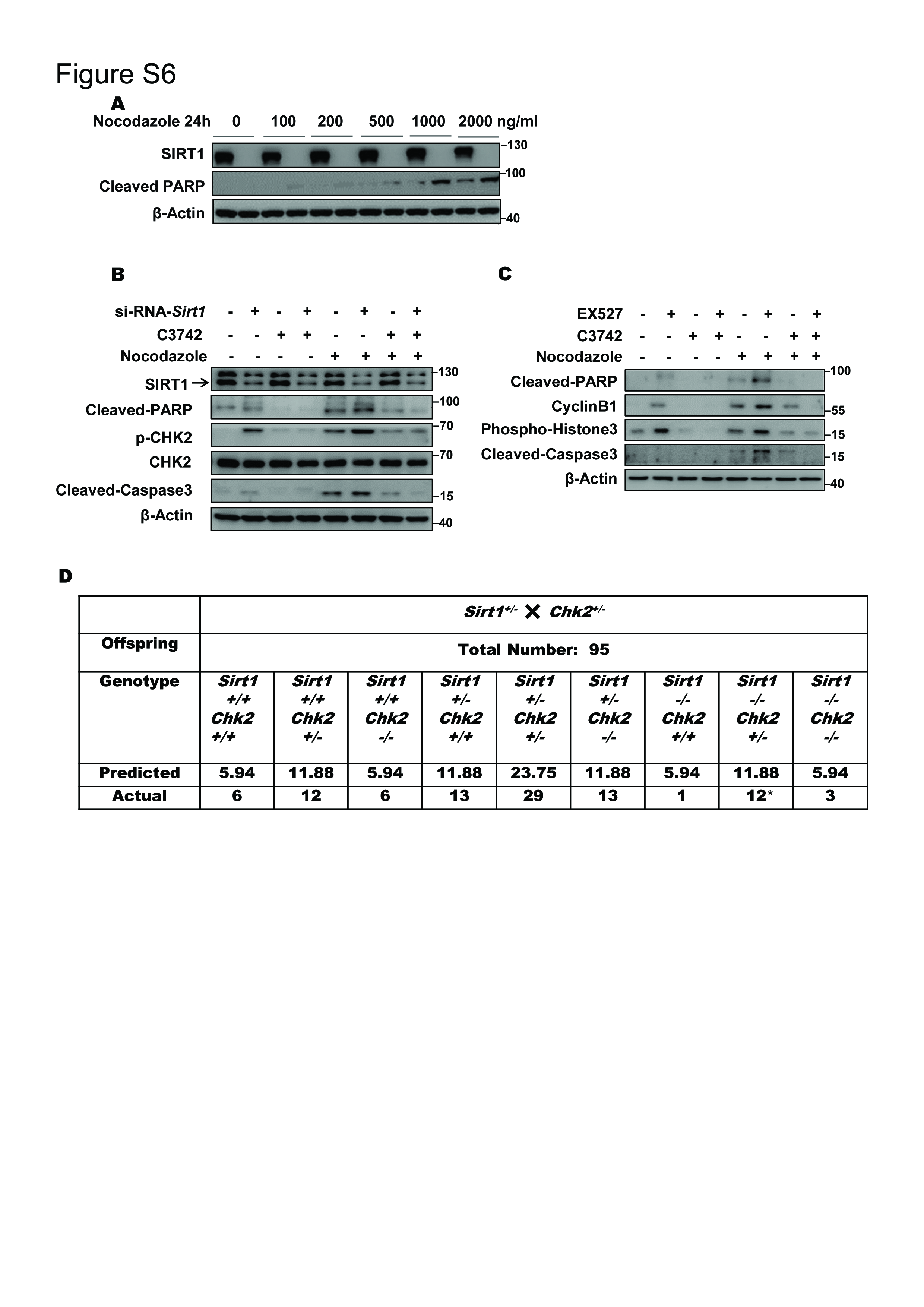
(A) Representative immunofluorescent images of an association of p-CHK2 positive staining (Thr68, red) with mitotic structures (GFP-H2B, green) and lagging chromosomes in HEK293 cells stably expressing control shRNA or sh*SIRT1*. Arrowhead indicates lagging chromosome (DAPI, blue). Scale bar, 50 μm. (B) HCT116 cells treated with indicated agents. DNA was stained with PI. Statistical differences were analyzed using Student’s t tests. Error bars represent ± SD. ***p < 0.001. (C) MEF cells were transfected with control or *CHK2* shRNA. SIRT1 and CHK2 levels were examined by Western blot analysis. (D) Representative FACS analysis of p-histone3 in the indicated genotypes treated with or without 100μM H_2_O_2_ for 1h (left) and quantification shown as the histogram(right). (E) Representative immunofluorescent images of cells as (C) treated with or without 100ng/ml of nocodazole for 12h (left) and quantification (right). *p < 0.05. **p < 0.01, ***p < 0.001. Related to Figure 5.

Figure S6 **CHK2 is essential for survival of SIRT1 deficiency.**

(A) *SIRT1^+/+^* and *SIRT1^−/−^* MEFs were incubated with media containing various concentrations of nocodazole for 24 hours and the cell lysates were measured with the indicated antibodies. (B) HELA cells transfected with the control and *Sirt1* siRNA were treated with or without 10μM of C3742 incubated in media containing nocodazole 50ng/ml for 24 hours or not. Cell lysates were subjected to Western blot analysis. (C) HEK293 cells treated with SIRT1 or CHK2 inhibitor as indicated were lysed and cell lysates were blotted and measured with the indicated antibodies. (D) Number of predicted and actual indicated genotypes of offspring from interbreeding of heterozygous mice. *p < 0.05, Related to Figure 6.
